# Editorial: The role of platinum-based antitumor prodrugs in medicinal inorganic chemistry

**DOI:** 10.3389/fchem.2023.1180682

**Published:** 2023-03-23

**Authors:** Nicola Margiotta, Mauro Ravera, Andrea Erxleben

**Affiliations:** ^1^ Dipartimento di Chimica, Università Degli Studi di Bari Aldo Moro, Bari, Italy; ^2^ Dipartimento di Scienze e Innovazione Tecnologica, Università Del Piemonte Orientale, Alessandria, Italy; ^3^ School of Biological and Chemical Sciences, University of Galway, Galway, Ireland

**Keywords:** cisplatin, oxaliplatin, carboplatin, prodrugs, medicinal inorganic chemistry

Medicinal Inorganic Chemistry is a relatively young area of research that developed after the great success of the coordination compound *cis*-diamminedichloridoplatinum(II), also known as cisplatin, that received FDA approval in 1978 for the treatment of testicular cancer and generated worldwide clinical interest in metal-based therapies. Since then, the use of metals in medicine is diverse and far-reaching, having been extended against bacterial, viral and parasitic infections as well as cardiovascular, age-related neurological diseases, diabetes, and arthritis. The great success of cisplatin in chemotherapy has encouraged the development of new platinum-based drugs, including second- and third-generation carboplatin and oxaliplatin (worldwide approval) as well as nedaplatin, heptaplatin, and lobaplatin (regional approval). Afterwards, platinum(IV) prodrugs were designed to overcome the problems generally associated to the traditional platinum(II) therapy such as many side effects and the development of chemoresistance, that partially limit the efficacy of platinum-based anticancer drugs. Since some of the side effects are caused by off-target reactions with biomolecules, the use of a Pt(IV) metal center is suggested due to its more inertness towards substitution reactions than Pt(II). Moreover, the hypoxic and low pH conditions surrounding tumor tissues and cells may selectively reduce Pt(IV) prodrugs with concomitant release of the two axial ligands and generation of the corresponding active Pt(II) antitumor species, increasing the selectivity. Other important clinical problems for platinum-based drugs are the lack of specificity and the low selectivity for tumor versus healthy cells. These problems could be overcome by rational selection of the axial ligands that allows to impart to the Pt(IV) prodrugs optimal hydro-/lipophilicity, red-ox stability, specific targeting and delivery, improved cellular uptake, and drug accumulation. To this aim, bioactive axial ligands have been widely explored to rationally design dual- or even multi-action Pt(IV) prodrugs that attack the cancer cell *via* two or more independent mechanisms.

This Research Topic of Frontiers in Chemistry—Medicinal and Pharmaceutical Chemistry highlights the problem of chemoresistance and the potential of Pt(IV) complexes to overcome resistance to classical Pt(II) drugs. It includes two review articles and two original research articles. The review by Mondal and Meeran discusses the role of non-coding RNAs in platinum-chemoresistance in lung cancer. An up to date account of the molecular mechanisms is given including the role of non-coding RNAs in the expression of drug transporters, DNA repair, control of cell division, epigenetic modifications, tumor microenvironment, epithelial-mesenchymal transition biomarker and transcription factor regulation, suggesting targeting non-coding RNAs as an interesting approach to enhance the efficacy of platinum cancer drugs. Huang et al. reviewed the literature on photoactivatable Pt(IV) prodrugs. Prodrugs that are activated on light irradiation can achieve higher selectivity than conventional prodrugs, as they allow for spatially and temporally controlled activation. The review focuses especially on the effect of the leaving, non-leaving and axial ligands on the photoactivity and photocytotoxicity. Gamal-Eldeen et al. showed that the oxygen carrier perftoran^®^ sensitizes resistant lung cancer cells to carboplatin. Co-treatment of cells with perftoran^®^ and carboplatin suppressed hypoxia pathway mediators and the drug resistance transporter MRP-2 and induced adduct formation between carboplatin and DNA. Moynihan et al. reported Pt(IV) prodrugs for the treatment of osteosarcoma. The authors synthesized a family of four carbohydrate-modified Pt(IV) complexes using click chemistry. The complexes were active in three different osteosarcoma cell lines. Interestingly, the prodrugs also showed activity against cancer stell cells.

While only a small number of Pt(IV) prodrugs have entered clinical trials to date [and those that did not show clear advantages over their Pt(II) counterparts], the research described in this Research Topic clearly indicates that the development of anticancer Pt(IV) complexes is an active and promising field.

